# Complete mitochondrial genome of *Ophichthus rotundus* (Anguilliformes: Ophichthidae)

**DOI:** 10.1080/23802359.2017.1303350

**Published:** 2017-03-23

**Authors:** Xiaojing Song, Wenqiao Tang

**Affiliations:** a Laboratory of Ichthyology, Shanghai Ocean University, Shanghai, China;; b Shanghai Key Laboratory of Marine Animal Taxonomy and Evolution, Shanghai, China

**Keywords:** *Ophichthus rotundus*, mitochondrial genome, phylogenetic analysis, Anguilliformes: Ophichthidae

## Abstract

The complete mitochondrial genome of *Ophichthus rotundus* was sequenced in this study. The genome sequence is 17,785 bp in length, comprising 13 protein-coding genes, 22 tRNA genes, 2 rRNA genes, and 2 control regions. Overall base composition is 30.59% A, 24.94% T, 17.01% G, and 27.46% C. The result of phylogenetic analysis indicates that *O. rotundus* mitogenome is close to that of *M. maculosus*, which are nested within the family Ophichthidae.

The order Anguilliformes contains 19 families, 159 genera, and about 938 species, most of which are marine origin. The Ophichthidae is a family of Anguilliformes, whose distinguishing features are posterior nostril usually within or piercing upper lip; median supraorbital pore in frontal sensory canal; caudal fin absent; pectoral fins present or absent; vertebrae 110–270 (Nelson et al. 2016). *Ophichthus rotundus* belongs to the family Ophichthidae, which distributes in Northwest Pacific, mainly including East China Sea and west coast of Korea (Zhang 2010). In this study, the complete mitochondrial genome of *O. rotundus* was determined. Fish sample was collected from the Changjiang Estuary (30°51′16.24″N, 121°54′46.51″E), Shanghai, China. The specimen is stored in the Laboratory of Ichthyology, Shanghai Ocean University, with an accession number 20160421. The sample DNA is available on request.

Mitochondrial DNA is a maternally inherited circular genome that serves important functions in metabolism and population genetics (Boore 1999). The complete mitogenome of *O. rotundus* (GenBank accession number: KY081397) is 17,785 bp in length, with overall base composition of 30.59% A, 24.94% T, 17.01% G, and 27.46% C. With the exception of two control regions, the genome content of *O. rotundus* includes 2 rRNA, 22 tRNA, and 13 protein-coding genes, plus the putative control region, as found in other vertebrates (Inoue et al. 2004). The original arrangement of protein-coding genes of order Anguilliformes is *nad1*–*nad2*–*cox1*–*cox2*–a*tp*8–*atp6*–*cox3*–*nad3*–*nad4L*–*nad4*–*nad5*–*nad6*–*cob*. The mitogenome of *O. rotundus* exhibits the translocation of *nad6*: *nad1*–*nad2*–*cox1*–*cox2*–*atp8*–*atp6*–*cox3*–*nad3*–*nad4L*–*nad4*–*nad5*–*cob*–*nad6* (Shen et al. 2014). Apart from the *ND6* gene and eight tRNA genes (*tRNA-Gln*, *Ala*, *Asn*, *Cys*, *Tyr*, *Ser*, *Glu*, and *Pro*) encoded on the L-strand, most genes are on the H-strand. 12 of 13 protein-coding genes start with a common initiation codon ATG, while *COI* utilizes GTG. *ND1*, *COI*, A*TP8*, *ND4L*, and *ND6* end with TAA; *ATP6* and *COIII* with TA– (incomplete stop codon); *ND2*, *COII*, *ND3*, and *ND4* with T–– (incomplete stop codon); *ND5* and *Cytb* with TAG.

To investigate the phylogenetic relationship among the order Anguilliformes, we downloaded the mitochondrial genome sequences of 12 currently available species of Anguilliformes, including *Anguilla australis* (AP007235), *A. obscura* (AP007247), *Coloconger cadenati* (AP010863), *Facciolella oxyrhyncha* (AP010866), *Heteroconger hassi* (AP010859), *Hoplunnis punctata* (AP010865), *Ilyophis brunneus* (AP010848), *Myrichthys maculosus* (AP010862), *Nessorhamphus ingolfianus* (AP010850), *Nettastoma parviceps* (AP010864), *Ophisurus macrorhynchos* (AP002978), and *Paraconger notialis* (AP010860), together with an African lungfish *Protopterus annectens* (NC018822) as an outgroup species. The phylogenetic tree was constructed using MEGA6 (Tamura et al. 2013) for neighbour-joining method. Tree topology was evaluated by 1000 bootstrap replicates. The result indicates that *O. rotundus* mitogenome is close to that of *M. maculosus*, which are nested within the family Ophichthidae (Figure 1).

**Figure 1.**
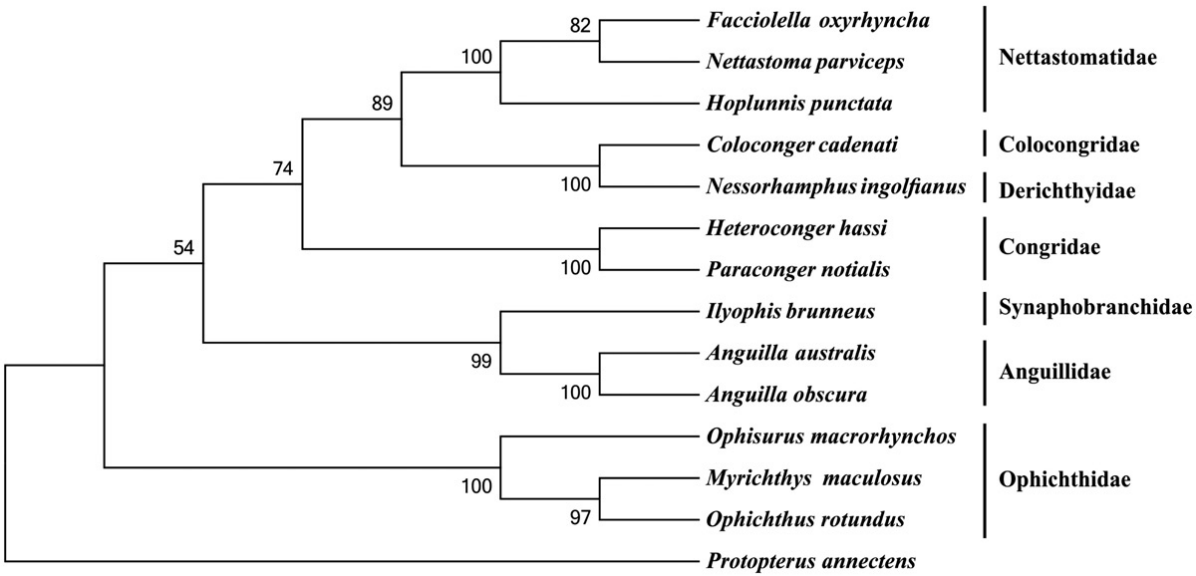
Phylogenetic tree of order Anguilliformes, with an African lungfish *Protopterus annectens* as an outgroup. The topology of phylogenetic tree was inferred from neighbour-joining method.

## References

[CIT0001] Boore JL. 1999 Animal mitochondrial genomes. Nucleic Acids Res. 27:1767–1780. 10101183 10.1093/nar/27.8.1767 PMC148383

[CIT0002] Inoue JG, Miya M, Tsukamoto K, Nishida M. 2004 Mitogenomic evidence for the monophyly of elopomorph fishes (Teleostei) and the evolutionary origin of the leptocephalus larva. Mol Phylogenet Evol. 32:274–286. 15186813 10.1016/j.ympev.2003.11.009

[CIT0003] Nelson JS, Grande TC, Wilson MVH. 2016 Fishes of the world. 5th ed New York (NY): John Wiley and Sons, Inc.

[CIT0004] Shen X, Tian M, Meng XP, Cheng HL, Yan BL. 2014 Eels mitochondrial protein-coding genes translocation and phylogenetic relationship analyses. Acta Oceanologica Sinica. 36:73–81.

[CIT0005] Tamura K, Stecher G, Peterson D, Filipski A, Kumar S. 2013 MEGA6: molecular evolutionary genetics analysis version 6.0. Mol Biol Evol. 30:2725–2729. 24132122 10.1093/molbev/mst197 PMC3840312

[CIT0006] Zhang 2010 Fauna Sinica, Osteichthyes, Anguilliformes & Notacanthiformes. Beijing: Science Press.

